# Supplementary material for: Acinetobacter baumannii K106 and K112: Two Structurally and Genetically Related 6-Deoxy-l-talose-Containing Capsular Polysaccharides
Source: Int J Mol Sci. 2021 May 26;22(11):5641. doi: 10.3390/ijms22115641 (PMC8199413; doi:10.3390/ijms22115641)
Supplement: Supplementary file 1 [file ijms-22-05641-s001.zip › ijms-1231257-supplementary.pdf]

Acinetobacter baumannii K106 and K112: Two structurally and genetically related 6-deoxy-L-talose-containing capsular polysaccharides

Anastasiya A. Kasimova [a], Nikolay P. Arbatsky [a], Ruth M. Hall [b], Jacob Tickner [c], Johanna J. Kenyon [c], Michael M. Shneider [d], Alina A. Dzhaparova [a,e], Alexander S. Shashkov [a], Alexander O. Chizhov [a *], Anastasiya V. Popova [f], Yuriy A. Knirel [a]

[a] N. D. Zelinsky Institute of Organic Chemistry, Russian Academy of Sciences, Moscow, Russia; nastia-kasimova979797@mail.ru (A.A.K.); nikolay.arbatsky@gmail.com (N.P.A.); shash@ioc.ac.ru (A.S.S); chizhov@ioc.ac.ru (A.O.C.); alina98d@gmail.com (A.A.D.); yknirel@gmail.com (Y.A.K.)

[b] School of Life and Environmental Sciences, The University of Sydney, Sydney, Australia; ruth.hall@sydney.edu.au (R.M.H.)

[c] School of Biomedical Sciences, Faculty of Health, Queensland University of Technology, Brisbane, Australia; j.tickner@uq.edu.au (J.T); johanna.kenyon@qut.edu.au (J.J.K.)

[d] M. M. Shemyakin and Yu. A. Ovchinnikov Institute of Bioorganic Chemistry, Russian Academy of Sciences, Moscow, Russia; mm_shn@mail.ru (M.M.S)

[e] D. I. Mendeleev University of Chemical Technology of Russia, Moscow, Russia; alina98d@gmail.com (A.A.D.)

[f] State Research Center for Applied Microbiology and Biotechnology, Obolensk, Moscow Region, Russia; popova_nastya86@mail.ru (A.V.P.)

* Correspondence: chizhov@ioc.ac.ru

a.

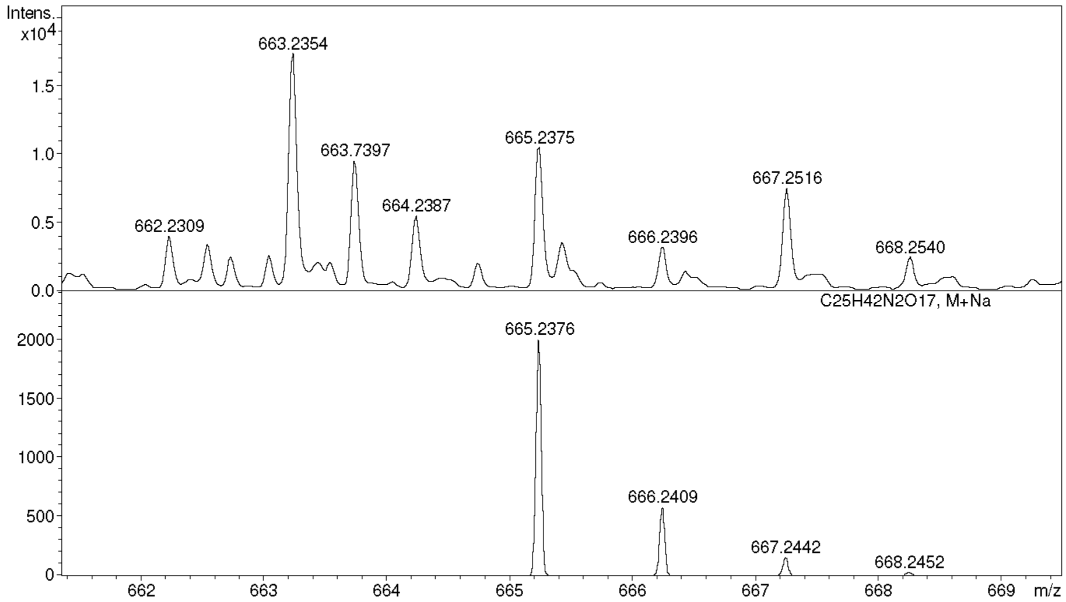

b.

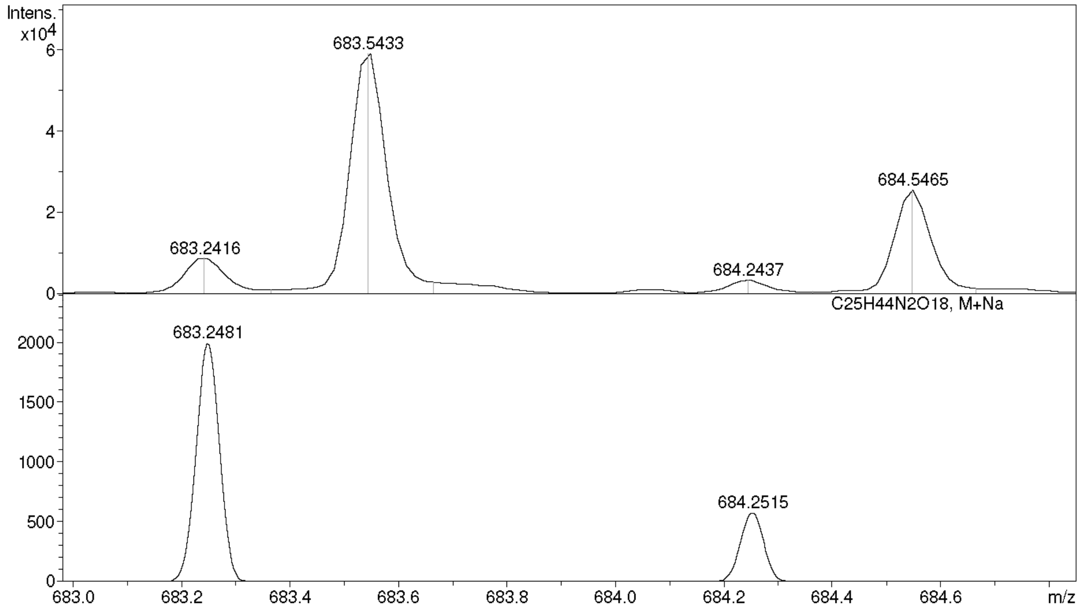

Figure S1, continued.

c.

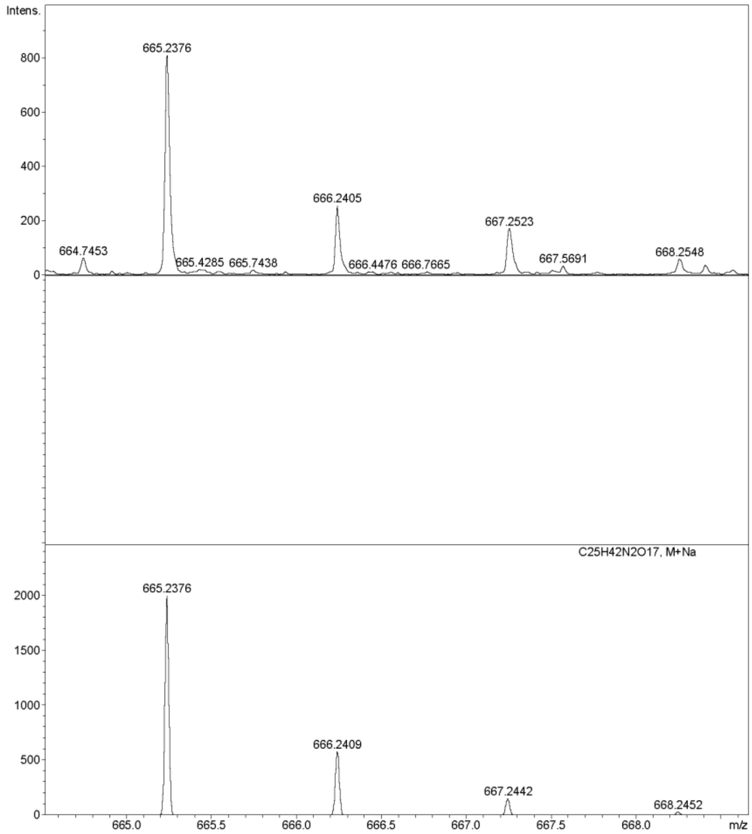

d.

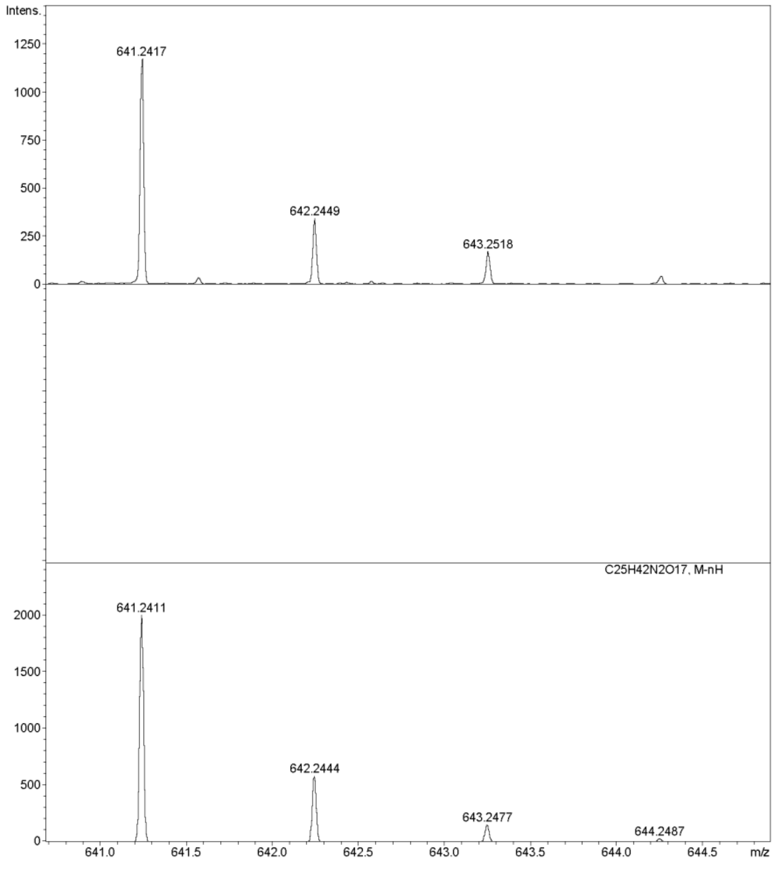

Figure S1, continued.

e.

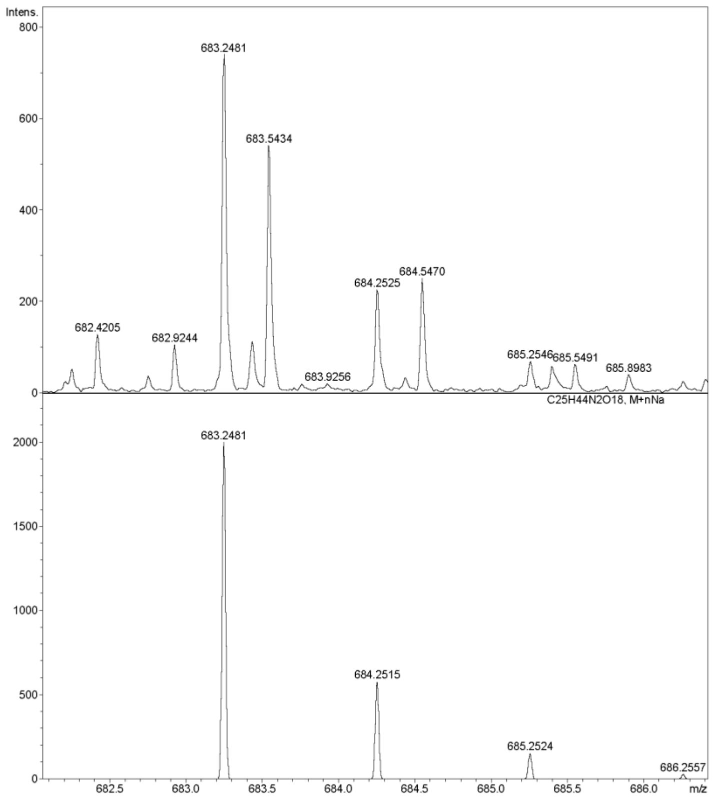

f.

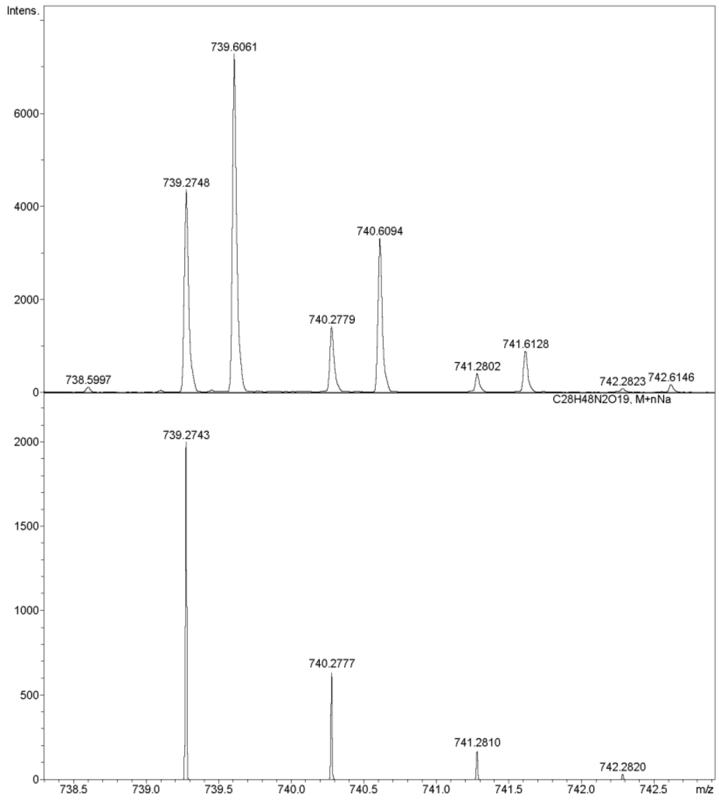

Figure S1, continued.

g.

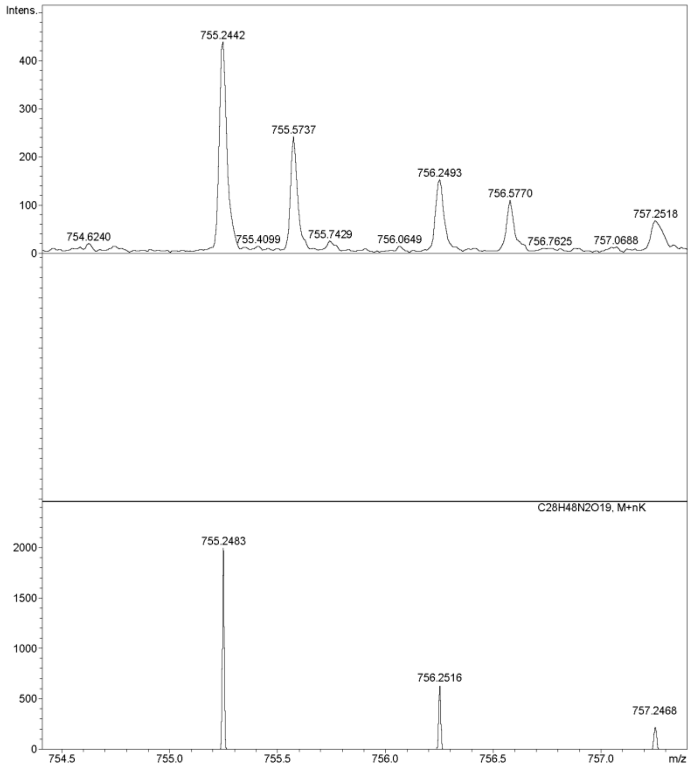

h.

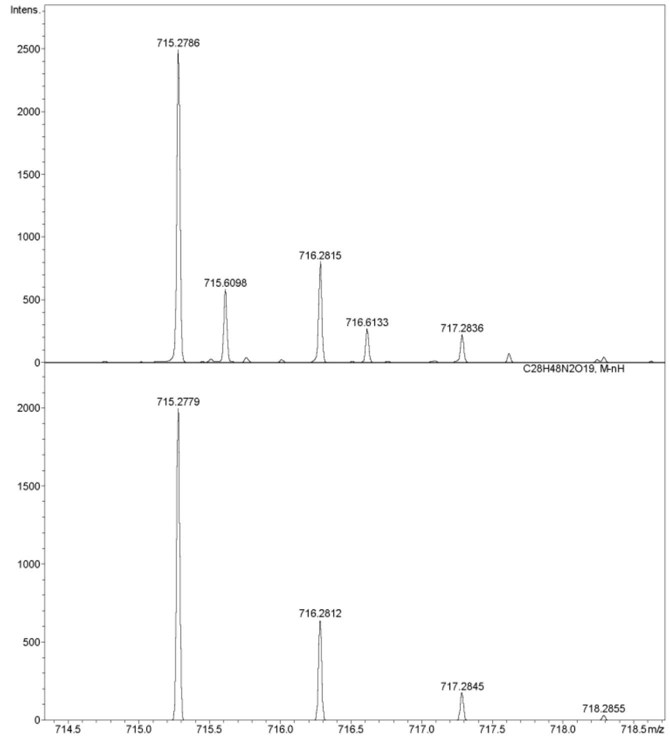

Figure S1. High-resolution, electrospray ionization mass spectra (HR ESI MS) of oligosaccharides OS1, OS2, and OS3. a. OS1, positive ion mode, $[M+Na]^+$. b. OS1 hydrate, positive ion mode, $[M+Na]^+$. c. OS2, positive ion mode, $[M+Na]^+$. d. OS2, negative ion mode, $[M–H]^–$. e. OS2 hydrate, positive ion mode, $[M+Na]^+$. f. OS3, positive ion mode, $[M+Na]^+$. g. OS3, positive ion mode, $[M+K]^+$. h. OS3, negative ion mode, $[M–H]^–$.

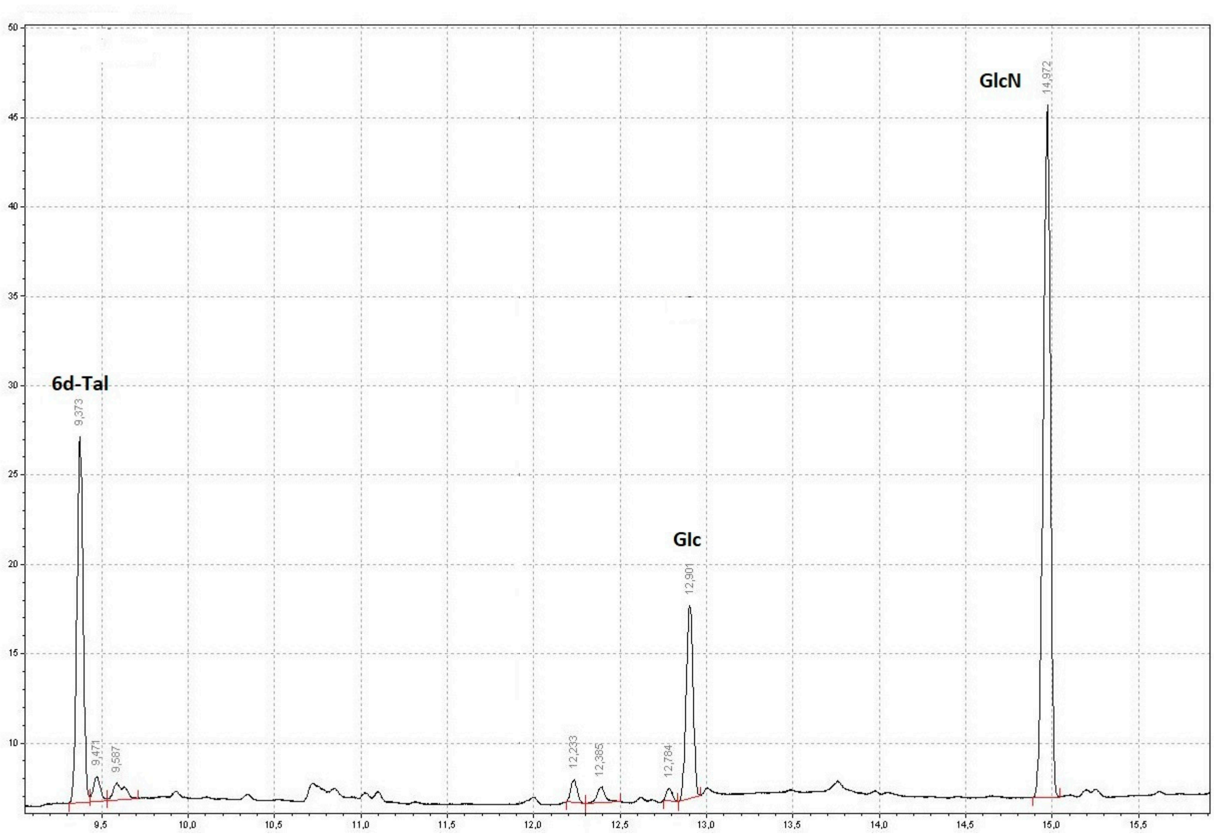

Figure S2. GLC chromatogram of sugar analysis of the K106 CPS of *A. baumannii* 48-1789**.**

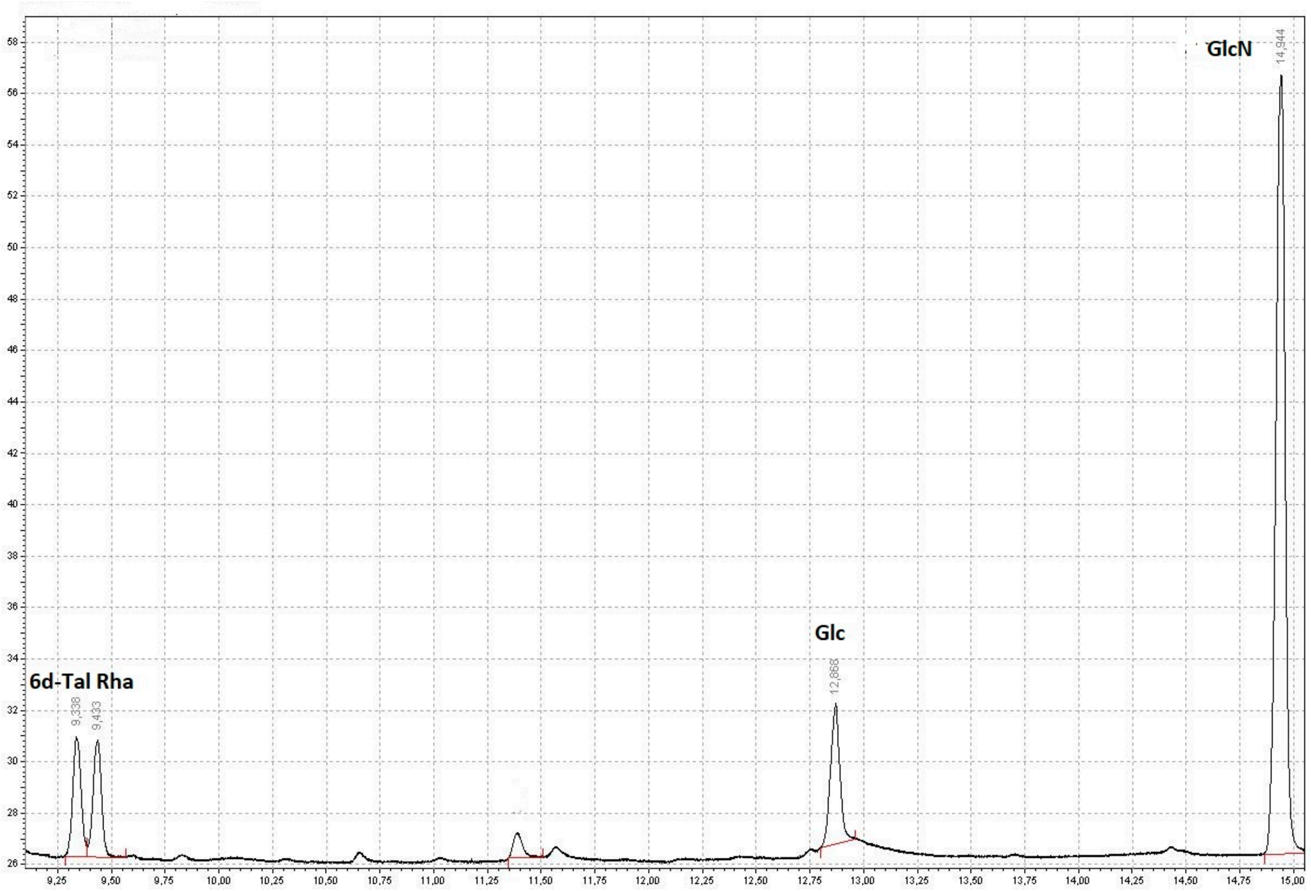

Figure S3. GLC chromatogram of sugar analysis of the K112 CPS of *A. baumannii* MAR24**.**

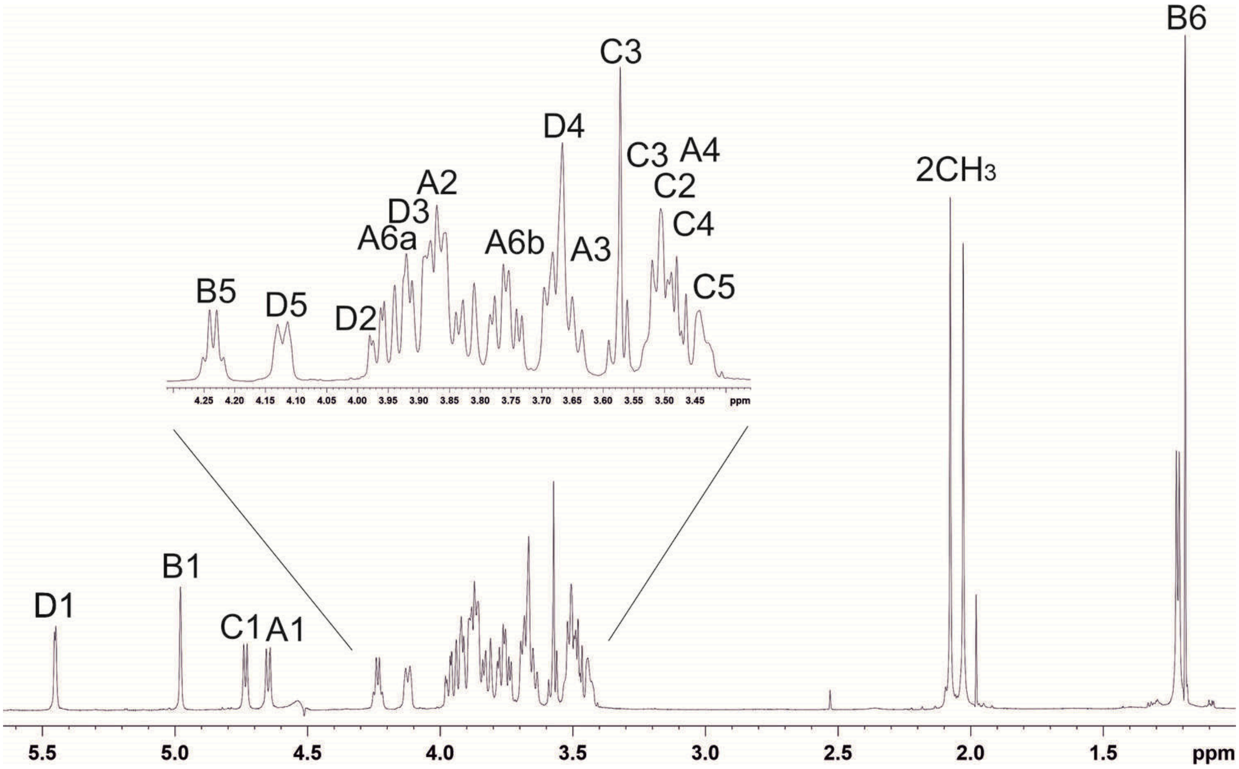

Figure S4. Part of ^{1}H NMR spectrum of the K106 CPS of *A. baumannii* 48-1789. Numbers refer to H atoms in sugar residues denoted by letters as indicated in Table 1.

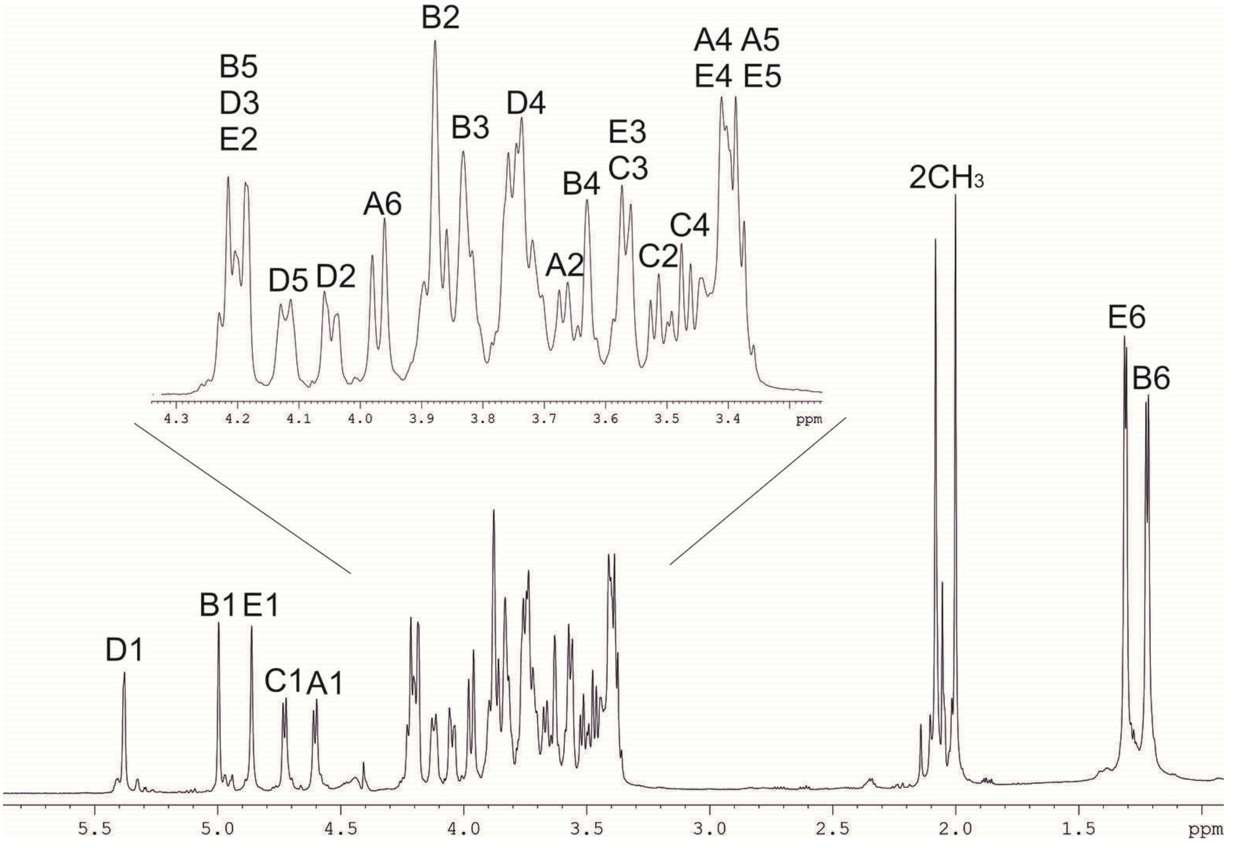

Figure S5. Part of ^{1}H NMR spectrum of the K112 CPS of *A. baumannii* MAR24. Numbers refer to H atoms in sugar residues denoted by letters as indicated in Table 2.

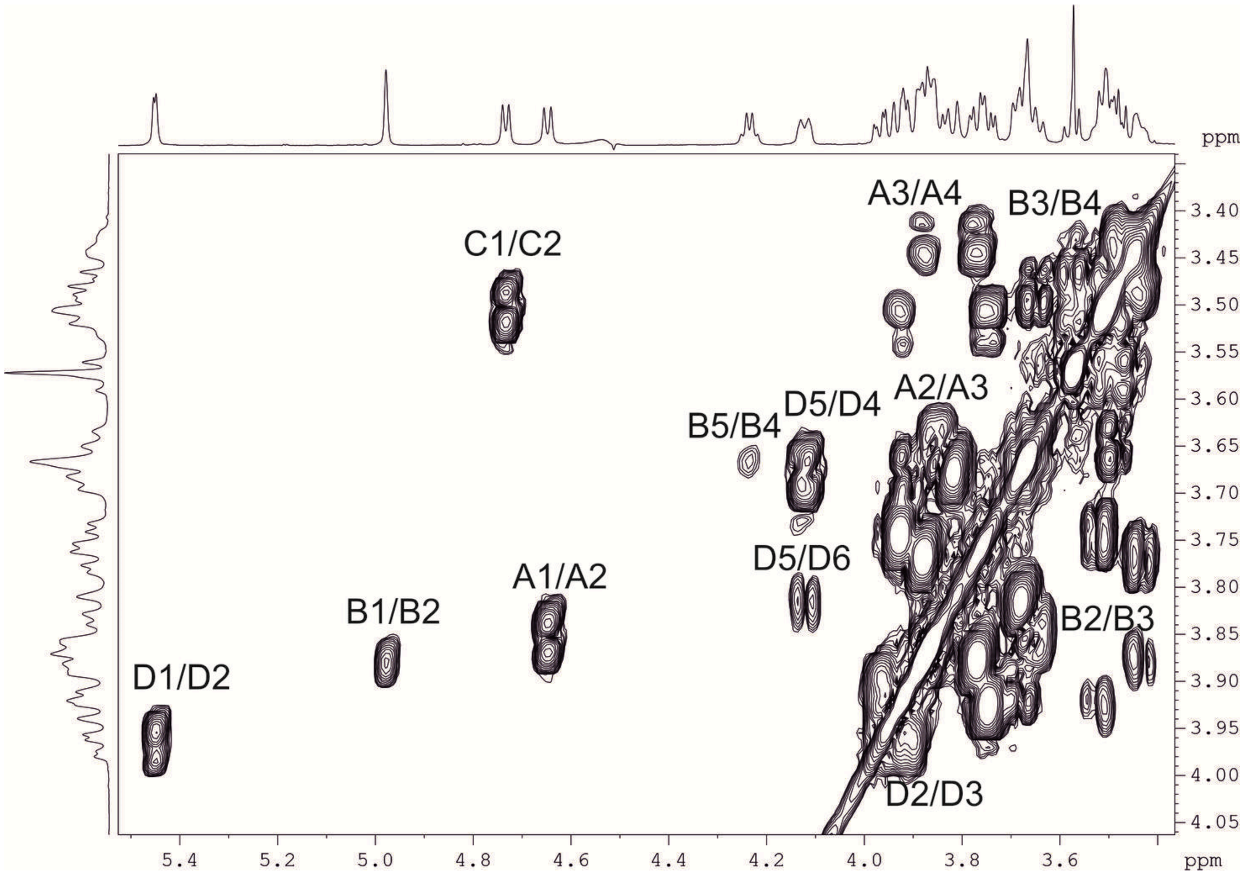

Figure S6. Part of a two-dimensional ^{1}H, ^{1}H COSY spectrum of the K106 CPS of *A. baumannii* 48-1789. The corresponding parts of the ^{1}H and ^{1}H NMR spectra are shown along the vertical and horizontal axes. Numbers refer to H/H pairs in sugar residues denoted by letters as indicated in Table 1.

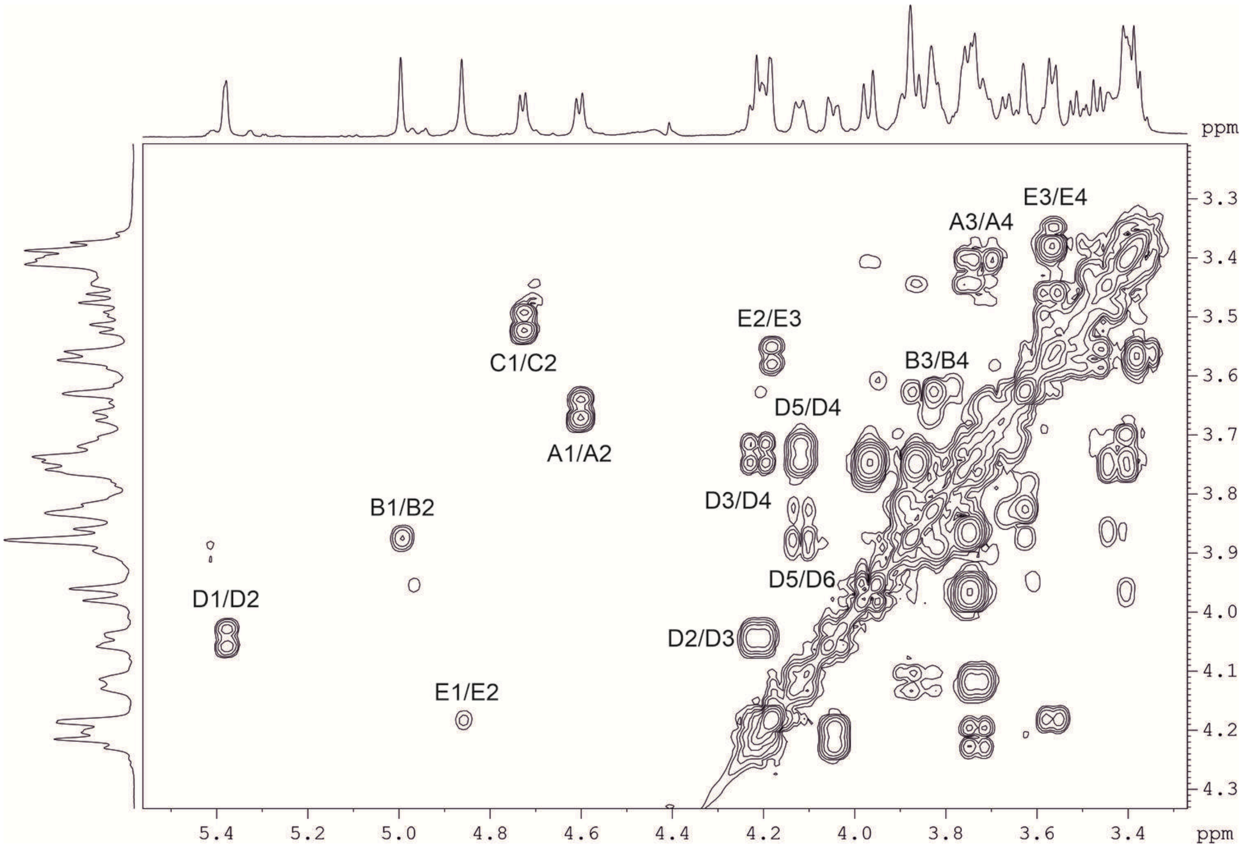

Figure S7. Part of a two-dimensional ^{1}H, ^{1}H COSY spectrum of the K112 CPS of *A. baumannii* MAR24. The corresponding parts of the ^{1}H and ^{1}H NMR spectra are shown along the vertical and horizontal axes. Numbers refer to H/H pairs in sugar residues denoted by letters as indicated in Table 2.

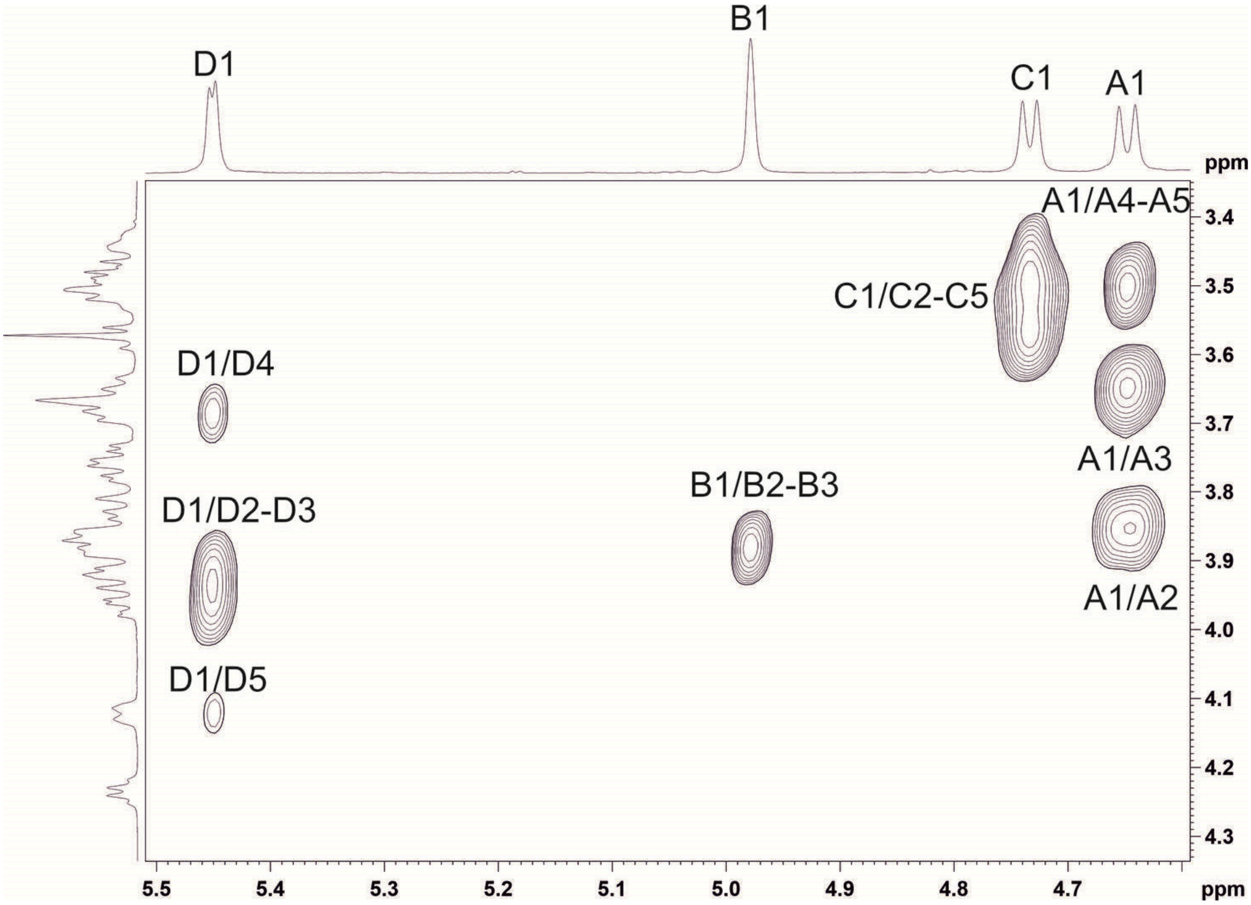

Figure S8. Part of a two-dimensional ^{1}H, ^{1}H TOCSY spectrum of the K106 CPS of *A. baumannii* 48-1789. The corresponding parts of the ^{1}H and ^{1}H NMR spectra are shown along the vertical and horizontal axes. Numbers refer to H/H pairs in sugar residues denoted by letters as indicated in Table 1.

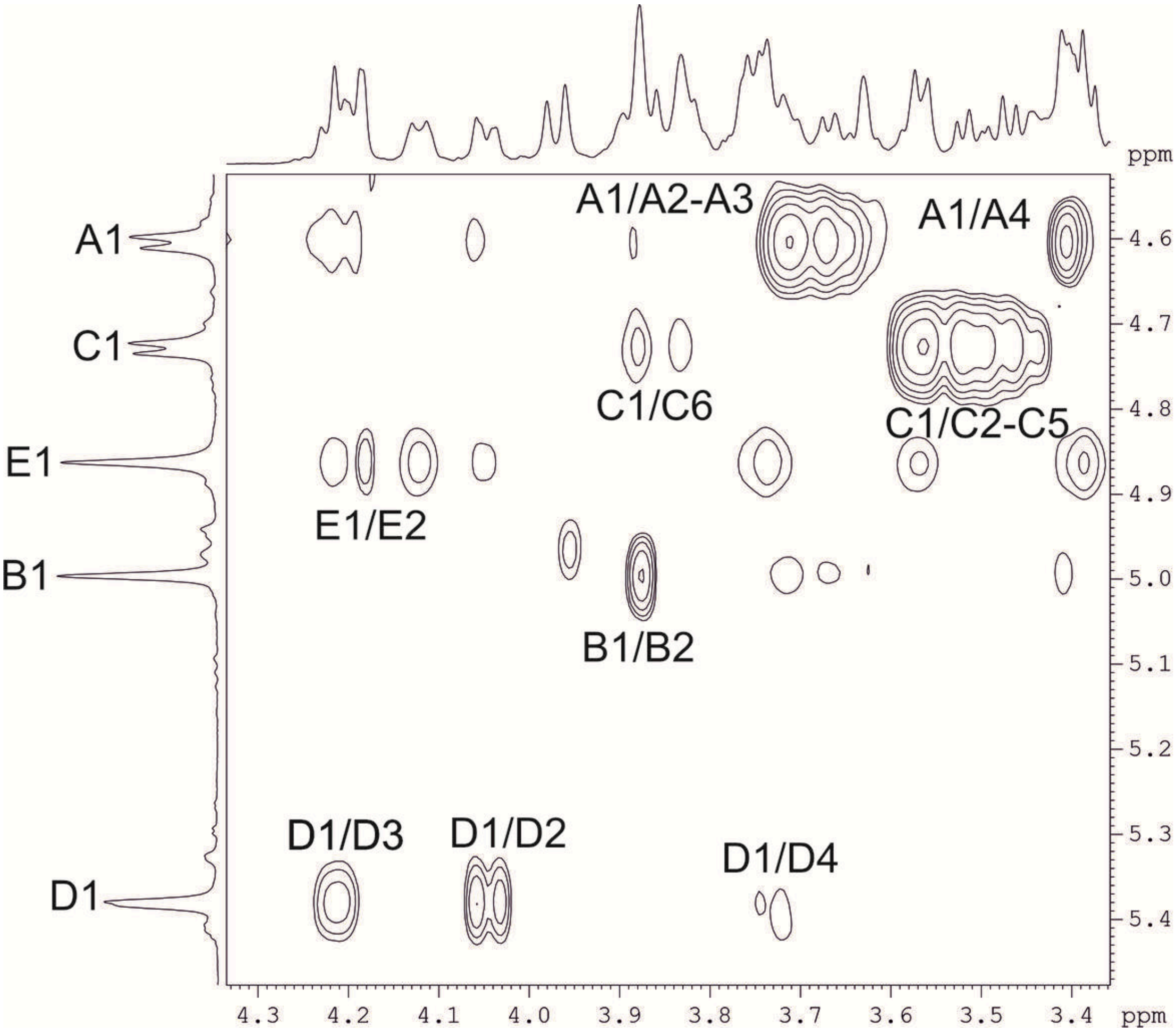

Figure S9. Part of a two-dimensional ^{1}H, ^{1}H TOCSY spectrum of the K112 CPS of *A. baumannii* MAR24. The corresponding parts of the ^{1}H and ^{1}H NMR spectra are shown along the vertical and horizontal axes. Numbers refer to H/H pairs in sugar residues denoted by letters as indicated in Table 2.

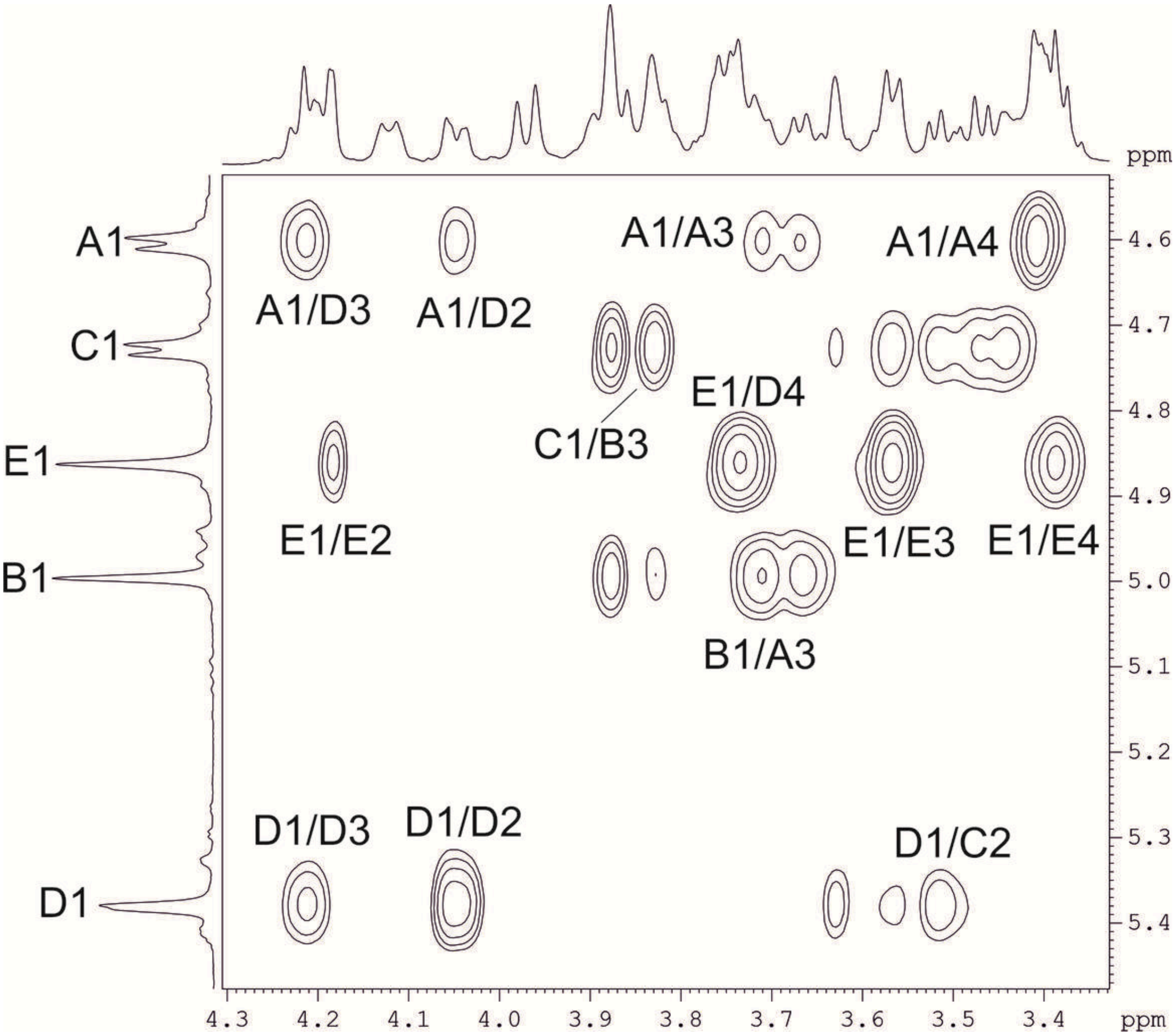

Figure S10. Part of a two-dimensional ^{1}H, ^{1}H ROESY spectrum of the K112 CPS of *A. baumannii* MAR24. The corresponding parts of the ^{1}H and ^{1}H NMR spectra are shown along the vertical and horizontal axes. Numbers refer to H/H pairs in sugar residues denoted by letters as indicated in Table 2.

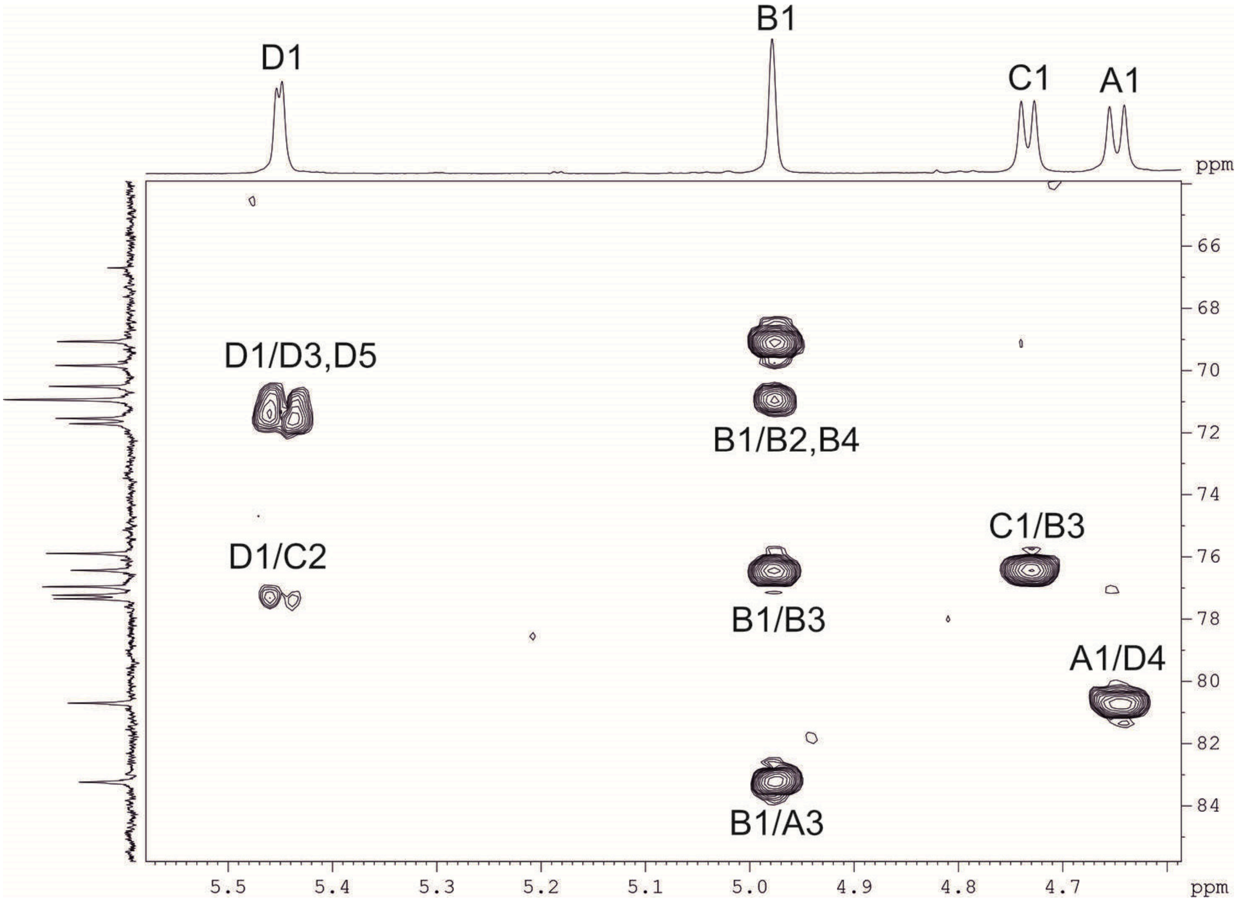

Figure S11. Part of a two-dimensional ^{1}H, ^{13}C HMBC spectrum of the K106 CPS of *A. baumannii* 48-1789. The corresponding parts of the ^{1}H and ^{13}C NMR spectra are shown along the horizontal and vertical axes, respectively. Numbers refer to H/C pairs in sugar residues denoted by letters as indicated in Table 1.

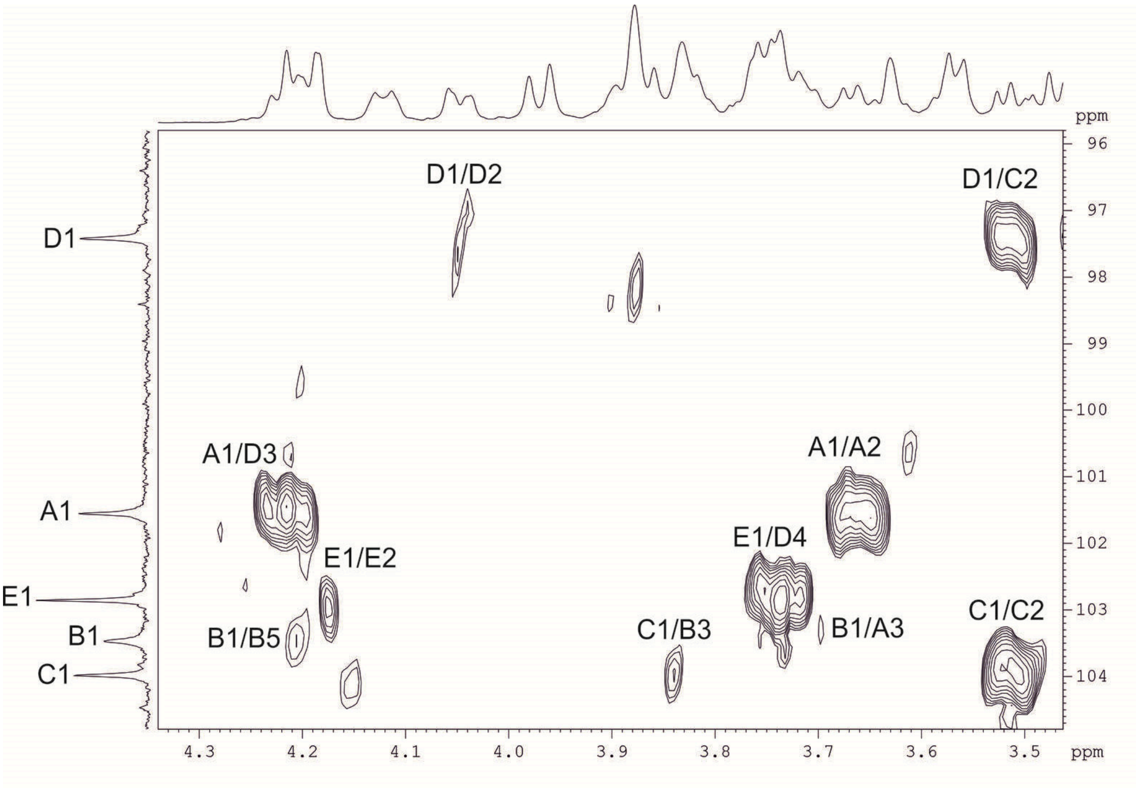

Figure S12. Part of a two-dimensional ^{1}H, ^{13}C HMBC spectrum of the K112 CPS of *A. baumannii* MAR24. The corresponding parts of the ^{1}H and ^{13}C NMR spectra are shown along the horizontal and vertical axes, respectively. Numbers refer to C/H pairs in sugar residues denoted by letters as indicated in Table 2.

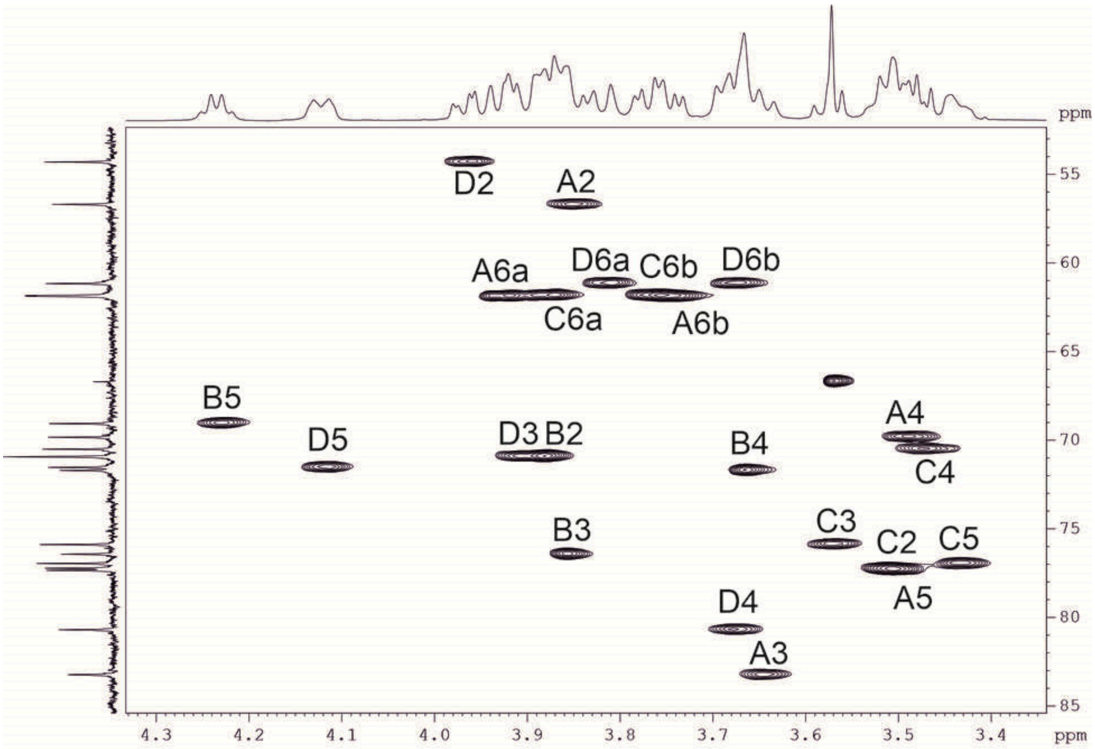
D2
A2
A6a
D6a
C6b
D6b
C6a
A6b
B5
D5
D3 B2
B4
A4
C4
B3
C3
C2
C5
A5
D4
A3
ppm
ppm

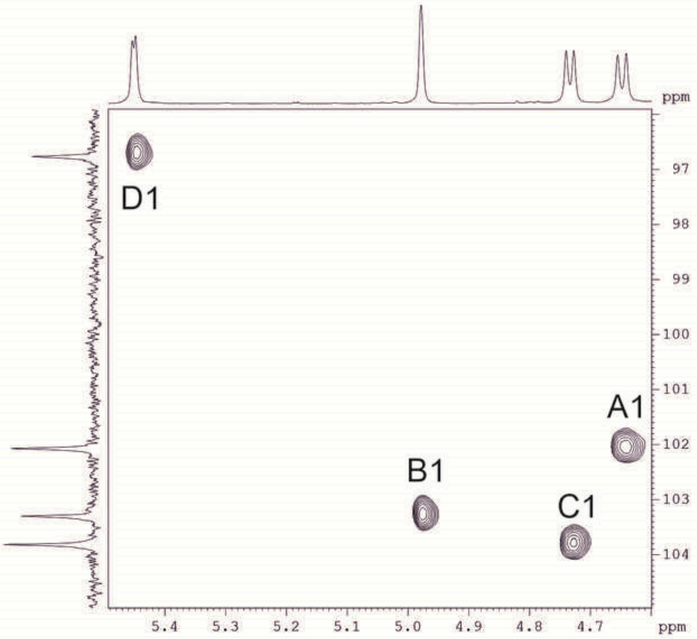
D1
A1
B1
C1
ppm
ppm

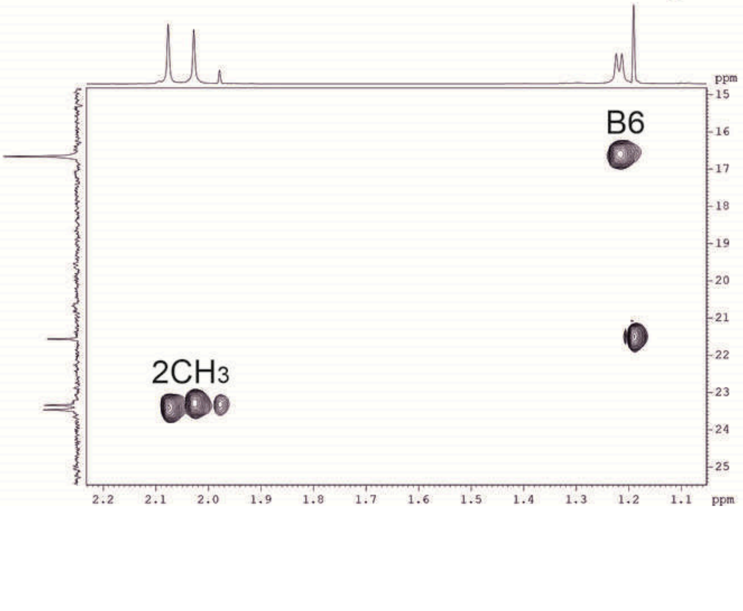
B6
2CH3
ppm
ppm

Figure S13. Parts of a two-dimensional ^{1}H, ^{13}C HSQC spectrum of the K106 CPS of *A. baumannii* 48-1789. The corresponding parts of the ^{1}H and ^{13}C NMR spectra are shown along the horizontal and vertical axes, respectively. Numbers refer to C/H pairs in sugar residues denoted by letters as indicated in Table 1.

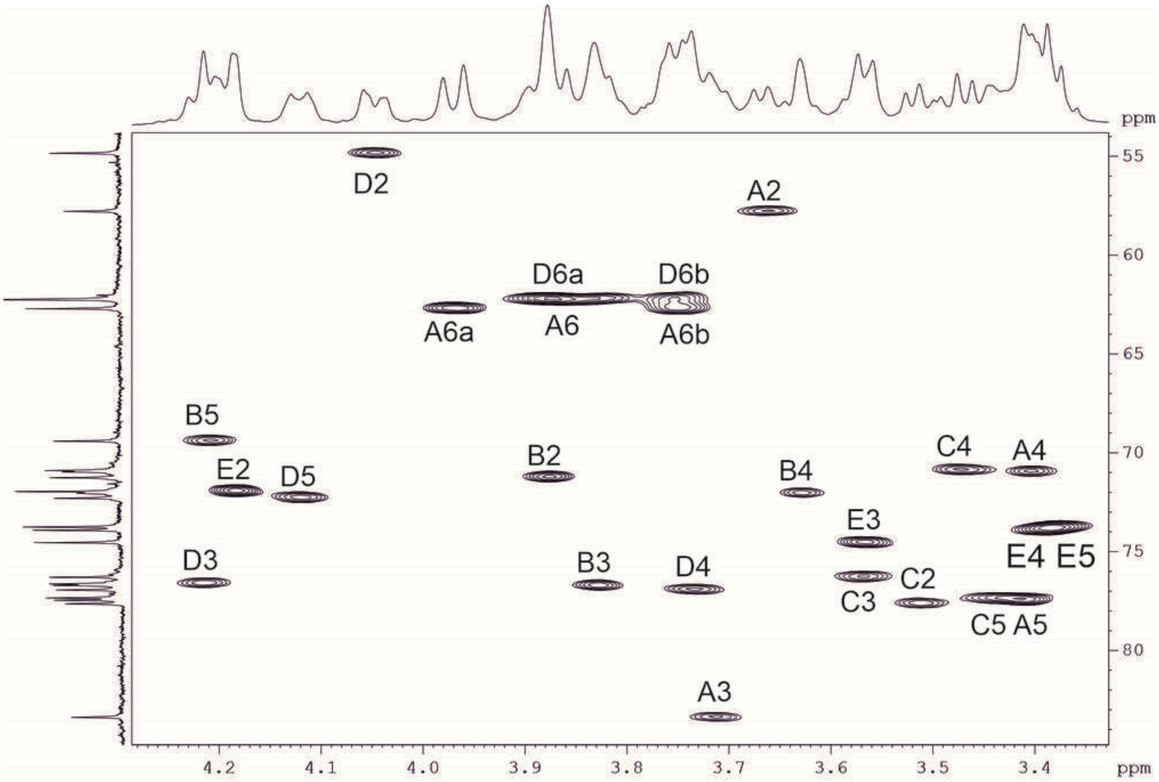
D2
A2
D6a
D6b
A6a
A6
A6b
B5
B2
C4
A4
E2
D5
B4
E3
E4 E5
D3
B3
D4
C2
C3
C5 A5
A3
ppm
ppm

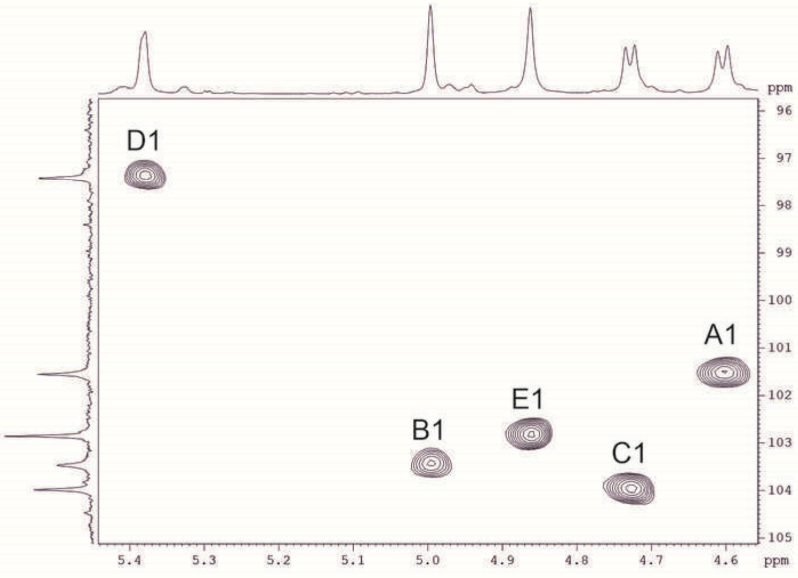
D1
A1
E1
B1
C1
ppm
ppm

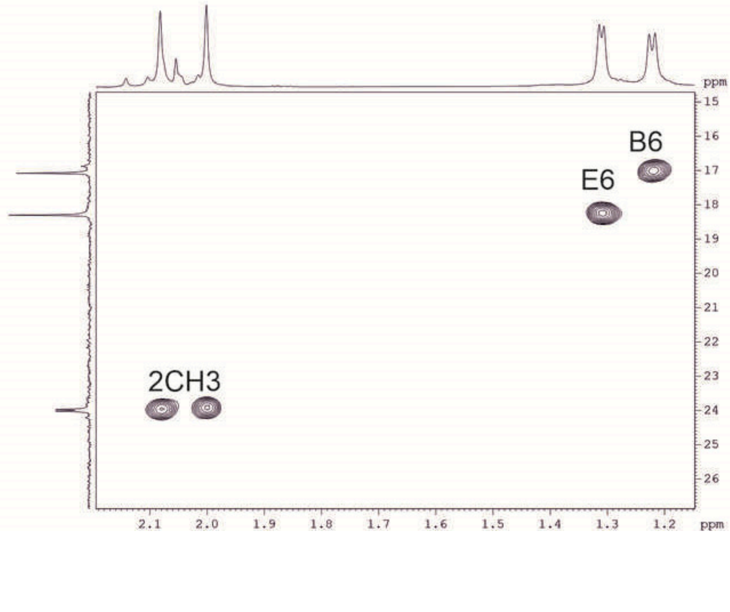
B6
E6
2CH3
ppm
ppm

Figure S14. Parts of a two-dimensional ^{1}H, ^{13}C HSQC spectrum of the K112 CPS of *A. baumannii* MAR24. The corresponding parts of the ^{1}H and ^{13}C NMR spectra are shown along the horizontal and vertical axes, respectively. Numbers refer to C/H pairs in sugar residues denoted by letters as indicated in Table 2.

In the reference list below, papers concerning identification, isolation and biosynthesis of 6-deoxy-L(D)-talose are presented.

References.

1. Knirel, Y. A.; Qian, C.; Senchenkova, S. N.; Guo, X.; Shashkov, A. S.; Chizhov, A. O.; Perepelov, A. V.; Liu, B. Structure of the β-L-fucopyranosyl phosphate-containing *O*-specific polysaccharide of *Escherichia coli* O84. *Internat. J. Biol. Macromol.* **2016**, 88, 578—585.
2. Maki, M.; Jarvinen, N.; Rabina, J.; Maaheimo, H.; Mattila, P.; Renkonen, R. Cloning and functional expression of a novel GDP-6-deoxy-D-talose synthetase from *Actinobacillus actinomycetemcomitans*. *Glycobiology* **2003**, 13, 295—303.
3. Suzuki, N.; Nakano, Y.; Yoshida, Y.; Nezu, T.; Terada, Y.; Yamashita, Y.; Koga, T. Guanosine diphosphate-4-keto-6-deoxy-D-mannose reductase in the pathway for the synthesis of GDP-6-deoxy-D-talose in *Actinobacillus actinomycetemcomitans*. *Eur. J. Biochem.*, **2002**, 269, 5963—5971.

4. Suzuki, N.; Nakano, Y.; Yoshida, Y.; Nakao, H.; Yamashita, Y.; Koga, T. Genetic analysis of the gene cluster for the synthesis of serotype a-specific polysaccharide antigen in *Actinobacillus actinomycetemcomitans*. *Biochim. Biophys. Acta — Gen. Struct. Exp.* **2000**, 1517, 135—138.

5. Katzenellenbogen, E.; Kocharova, N.A.; Zatonsky, G.V.; Bogulska, M.; Witkowska, D.; Shashkov, A.S.; Knirel, Y.A.; Romanowska, E. Structure of the *O*-specific polysaccharide of *Hafnia alvei* 23 having an oligosaccharide-phosphate repeating unit. *J. Carbohydr. Chem.* **1999**, 18, 545—558.

6. Zahringer, U.; Rettenmaier, H.; Moll, H.; Senchenkova, S.N.; Knirel, Y.A. Structure of a new 6-deoxy-alpha-D-talan from *Burkholderia* (*Pseudomonas*) *plantarii* strain DSM 6535, which is different from the *O*-chain of the lipopolysaccharide. *Carbohydr. Res.* **1997**, 300, 143-151.

7. Perry, M.B.; Maclean, L.M.; Brisson, J.R.; Wilson, M.E. Structures of the antigenic *O*-polysaccharides of lipopolysaccharides produced by *Actinobacillus actinomycetemcomitans* serotypes a, c, d and e. *Eur. J. Biochem*. **1996**, 242, 682—688.

8. Shibuya, N.; Amano, K.; Azuma, J.; Nishihara, T.; Kitamura, Y.; Nogychi, T.; Koga, T. 6-Deoxy-D-Talan and 6-deoxy-L-talan — Novel serotype-specific polysaccharide antigens from *Actinobacillus actinomycetemcomitans*. *J. Biol. Chem*. **1991**, 266, 16318—16323

9. Andersson, M.; Ratnayake, S.; Kenne, L.; Ericsson, L.; Stack, R.J. Structural studies of the exreacellular polysaccharide from *Butyrivibrio fibrisolvens* strain X6C61. *Carbohydr. Res*. **1993**, 246, 291—301.

10. Stack, R.J. Identification of 6-deoxy-D-talose in an extracellular polysaccharide produced by *Butyrivibrio fibrisolvens* strain X6C61. *Carbohydr. Res*. **1989**, 189, 281—288.
